# Administration of anti-ERMAP antibody ameliorates Alzheimer’s disease in mice

**DOI:** 10.1186/s12974-021-02320-x

**Published:** 2021-11-13

**Authors:** Haiyan Liu, Jin Zhao, Yujun Lin, Min Su, Laijun Lai

**Affiliations:** 1 Shandong Provincial Hospital Affiliated to Shandong First Medical University, Jinan, 250021 China; 2 Department of Allied Health Sciences, University of Connecticut, 1390 Storrs Road, Storrs, CT 06269 USA; 3 Department of Human Histology and Embryology, Tissue Engineering and Stem Cell Research Center, Guizhou Medical University, Guiyang, 550004 China; 4 University of Connecticut Stem Cell Institute, University of Connecticut, Storrs, CT USA

**Keywords:** Alzheimer’s disease, Amyloid-beta, ERMAP, T cells, Macrophage, Choroid plexus

## Abstract

**Background:**

Alzheimer’s disease (AD) is a devastating age-related neurodegenerative disorder and characterized by progressive loss of memory and cognitive functions, which are associated with amyloid-beta (Aβ) plaques. Immune cells play an important role in the clearance of Aβ deposits. Immune responses are regulated by immune regulators in which the B7 family members play a crucial role. We have recently identified erythroid membrane-associated protein (ERMAP) as a novel B7 family-related immune regulator and shown that ERMAP protein affects T cell and macrophage functions.

**Methods:**

We produced a monoclonal antibody (mAb) against ERMAP protein and then determined the ability of the mAb to affect cognitive performance and AD pathology in mice.

**Results:**

We have shown that the anti-ERMAP mAb neutralizes the T cell inhibitory activity of ERMAP and enhances macrophages to phagocytose Aβ in vitro. Administration of the mAb into AD mice improves cognitive performance and reduces Aβ plaque load in the brain. This is related to increased proportion of T cells, especially IFNγ-producing T cells, in the spleen and the choroid plexus (CP), enhanced expression of immune cell trafficking molecules in the CP, and increased migration of monocyte-derived macrophages into the brain. Furthermore, the production of anti-Aβ antibodies in the serum and the macrophage phagocytosis of Aβ are enhanced in the anti-ERMAP mAb-treated AD mice.

**Conclusions:**

Our results suggest that manipulating the ERMAP pathway has the potential to provide a novel approach to treat AD patients.

## Background

Alzheimer’s disease (AD) is a devastating neurodegenerative disease and the most common form of dementia of older adults [1]. There are over 34 million people who suffer from AD worldwide, and the number is expected to triple over the next 40 years [2]. Although intensive efforts have been made in treating AD, currently approved interventions for AD have shown only modest effects in modifying clinical symptoms, and none have shown effects on disease progression. Therefore, new strategies to study AD pathogenesis and treat AD are required.

AD is characterized by progressive loss of memory and cognitive functions [1, 3–6]. The cognitive decline is related to hallmark protein aggregates, amyloid-β (Aβ) plaques and neurofibrillary tangles, which can accelerate neuronal cell death, adversely affect synaptic function and eventually cause neuron loss [1, 3–7].

Although the brain has been traditionally considered immune privileged [8, 9], compelling data have suggested that the CNS is actually immunocompetent, and that neuroimmune communication plays an important role in CNS homeostasis and function, as well as brain repair, especially in pathological conditions including AD [9–15].

Immune cells are tightly regulated by stimulatory and inhibitory molecules. Among the immune regulators, B7 family members play a crucial role in regulating immune responses. The importance of the B7 family has been emphasized by the FDA approval of several drugs for the treatment of cancer and autoimmune disease by targeting the ligands or receptors of the B7 family members PD-L1/PD-1 and CTLA-4. Recognizing the importance of the B7 family in controlling immune responses, we performed a series of genome-wide database searches to find molecules that are homologous to known B7 family members. We have discovered that erythroid membrane-associated protein (ERMAP) shares sequence and structural similarities with the known B7 family members [16]. We have shown that the ERMAP receptor is expressed on T cells and macrophages [16]. ERMAP protein inhibits T cell functions and increases the generation of anti-inflammatory M2 macrophages, whereas anti-ERMAP antibody neutralizes the T cell inhibitory activity of ERMAP and enhances macrophage phagocytosis ability [16].

It has been reported that both T cells and macrophages are involved in AD development [5, 8, 17–28]. Since ERMAP can affect the functions of T cells and macrophages, we determined that ability of an anti-ERMAP monoclonal antibody (mAb) to affect AD development. We show here that administration of the anti-ERMAP mAb increases the functions of both T cells and macrophages, resulting in reduced Aβ plaque load and improved cognitive performance in AD mice.

## Methods

### Mice

3xTg-AD, APP/PS1, C57BL/6 mice were purchased from Jackson Laboratory. The mice were used in accordance with a protocol approved by the Institutional Animal Care and Use Committee of the University of Connecticut.

### Generation of ERMAP mAb

BALB/c mice were immunized with 50 μg human ERMAP-Ig protein emulsified in complete Freund's adjuvant (CFA) on day 0 and boosted on day 14 and day 21 in the same protein quantity in incomplete Freund's adjuvant (IFA). The mice were boosted with 50 μg ERMAP-Ig without IFA 3 times (days 28, 29, and 30). On day 31, the spleens were harvested from the immunized mice. Single-cell suspension of the splenocytes were fused to X63-Ag8.653 myeloma cells to produce hybridomas. ELISA was performed to identify the hybridomas that could produce anti-ERMAP mAbs reacting with ERMAP. The anti-ERMAP mAbs were further screened for the ability to neutralize the inhibitory activity of ERMAP-Ig on T cell proliferation and activation.

### Real-time qualitative reverse transcription polymerase chain reaction (RT-PCR) (qRT-PCR)

Total RNA was extracted from tissues or cells using a Nucleo Spin RNA II kit (Macherey-Nagel, Düren, Gemany). The RNA was converted into complementary DNA using High Capacity cDNA Reverse Transcription Kit (Invitrogen, USA). qRT-PCR was performed with the Power SYBR green master mix (Applied Biosystems, UK) using the 7500 real-time PCR system (Applied Biosystems, UK).

### Immunohistochemistry

The brain tissues were incubated in a fixative solution, embedded in OCT medium, snap-frozen, and subsequently cut into 6-µm sections that were incubated with the following primary antibodies: mouse anti-Aβ (clone 6E10,) and rabbit anti-GFAP (Biolegend, USA). After washing, the sections were incubated with fluorochrome-conjugated secondary antibody, counterstained with 4’,6’-diamidino-2-phenylindole (DAPI) and observed under a Nikon A1R Spectral Confocal microscope (Nikon, Kanagawa, Japan). To quantify the staining intensity, total cells and background fluorescence intensity were measured using ImageJ software (NIH, USA), and the intensity of specific staining was calculated as described [5].

### Flow cytometry analysis

Mice were transcardially perfused with phosphate-buffered saline (PBS) before tissue excision. Brains were dissected, and different brain regions were removed under a dissecting microscope. CP tissues were isolated from the lateral, third and fourth ventricles of the brain; and single-cell suspension was prepared as described [5, 22]. Spleens were mashed with the plunger of a syringe and treated with ammonium chloride potassium-lysing buffer to remove erythrocytes. Cells were stained with fluorochrome-conjugated antibodies directly or indirectly as described [29]. For intracellular staining, the cells were first permeabilized with a BD Cytofix/Cytoperm solution for 20 min at 4℃. The following antibodies were used: CD4, CD8, CD45, CD11b, F4/80, IFNγ, Ly6c, and SRA1 (BioLegend, San Diego, CA, or ThermoFisher Scientific). The samples were analyzed on an LSRFortessa X-20 Cell Analyzer (BD Biosciences). Data analysis was performed using FlowJo software (Ashland, OR).

### T cell proliferation assay

Murine CD3^+^ T cells were purified from C57BL/6 mice by an immunomagnetic system (Miltenyi, Auburn, CA). T cells were stimulated with anti-CD3 antibody (Biolegend) with ERMAP-Ig or control Ig in the presence of anti-ERMAP mAb or control Ab. Proliferative response was assessed by pulsing the culture with 1 µCi of [^3^H] thymidine (PerkinElmer, Inc., Downers Grove, IL) 12 h before harvest. [^3^H] thymidine incorporation was measured by liquid scintillation spectroscopy (PerkinElmer, Inc.).

### ELISA assay for Anti-Aβ40 or anti-Aβ42 antibody

Aβ40 or Aβ42 (Anaspec, USA) was coated on 96-well microplates overnight at 4 ℃, then blocked with blocking buffer (2% BSA + 5% goat serum in PBS) for 2 h at room temperature. The serum samples diluted into 1:1000 were added to the plates and incubated 2 h at room temperature. After washing, HRP-conjugated goat anti-mouse IgG (Biolegend) was added to the plates and incubated for 1 h. The reaction was developed by TMB substrate (Thermo Scientific, USA) and stopped with 0.1 N HCl. The microplate was read at 450 nm under a microplate reader (Bio-Tek, ELX800, USA). The antibody concentrations were calculated using a standard curve generated with known concentrations of anti-Aβ antibody.

### Soluble Aβ protein (sAβ) isolation and quantification

Brain parenchyma was dissected, snap-frozen and kept at −75 °C until homogenization. The samples were homogenized, and the supernatants were collected and detected for the concentrations of Aβ_1-40_ and Aβ_1-42_ by ELISA as described [22, 30].

### Western blot

Brain (cortex and hippocampus regions) was homogenized in RIPA lysis buffer containing protease inhibitors as described [31]. Equal amounts of sample protein were separated on Tris–HCl polyacrylamide SDS gels and transferred to polyvinylidene fluoride membranes. Blots were placed in blocking solution with 10% non-fat milk in PBS with 0.05% Tween-20 (PBS-T) for 1 h, followed by incubation with various primary antibodies with 5% non-fat milk in PBS-T for 3 h at room temperature or overnight at 4 °C. Primary antibodies include BACE1 (Santa Cruz), ADAM10 (Santa Cruz), ADAM17 (Millipore Sigma), Presenilin-1 (PS-1) (Novus Biologicals) and APP [to detect both full-length APP and C-terminal fragments (CTFs); CT695, ThermoFisher]. Blots were washed with PBS-T, incubated with horseradish peroxidase-conjugated secondary antibodies, and then developed with Super Signal® West Pico chemiluminescent Substrate (Thermo Scientific).

### Amyloid phagocytosis assay

HiLyte Fluor 647 Beta-amyloid (1–42) (Anaspec) was resuspended in Tris/EDTA (pH 8.2) at 20 mM and then incubated in the dark for 3 days at 37 °C to promote aggregation. Macrophages in suspension were pretreated in low serum medium as described [32]. The HiLyte Fluor 647 Beta-amyloid was added and incubated for 5 h. Cells were stained with macrophage markers; amyloid phagocytosis by the macrophages was determined by flow cytometry [30, 32].

### Barnes maze

Barnes maze was conducted as previously described [33, 34]. Briefly, each mouse was placed in the center of the maze and subjected to aversive stimuli. Mice were trained 4 training trials per day for 5 days, and a probe test was performed 24 h after the last training trial. The latency and number of errors were recorded for the training tail and probe test.

### Novel object recognition (NOR) test

A NOR test was conducted as previously described [33–35]. Briefly, mice were trained by allowing them to explore two identical objects placed at opposite ends of the arena for 10 min. 24 h later, mice were tested with one copy of the familiar object and one novel object of similar dimensions for 3 min. The time spent on exploring and sniffing of each object was recorded. The NOR index represents the percentage of time mice spent exploring the novel object.

### Statistical analysis

For comparing means of 2 groups, two‐tailed Student’s *t*‐test was used. For comparing means of multiple groups, significance was determined using one‐way ANOVA with Dunnett test. Differences with *P* < 0.05 were considered statistically significant.

## Results

### Anti-ERMAP mAb neutralizes the T cell inhibitory activity of ERMAP and enhances macrophage phagocytosis of Aβ

**Fig. 1:**
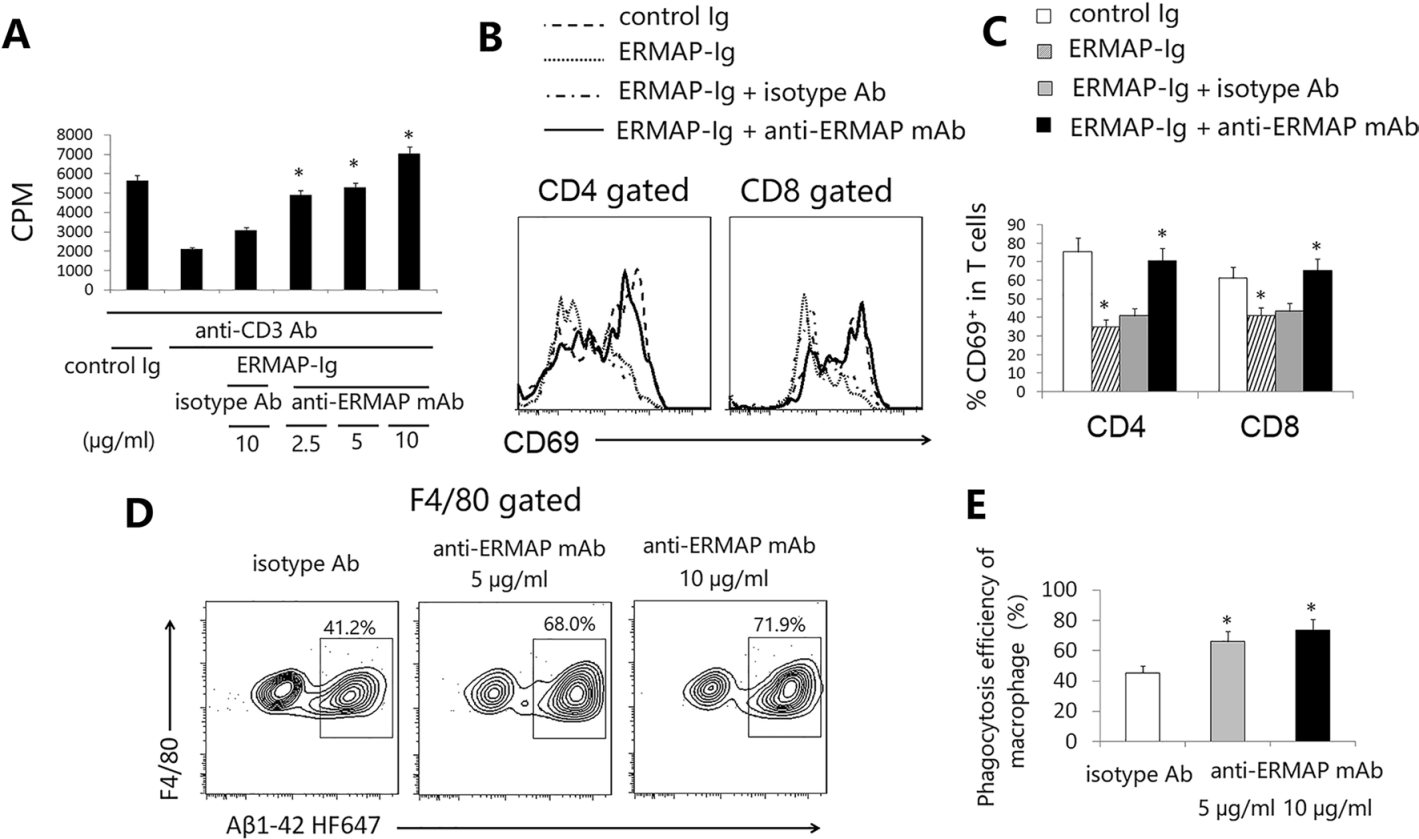
An anti-ERMAP mAb neutralizes T cell inhibitory activity of ERMAP-Ig and enhances macrophage phagocytosis of Aβ in vitro. **A** T cells were purified from splenocytes of C57BL/6 mice and cultured with anti-CD3 Ab (1 μg/ml) in the presence of control Ig or human ERMAP-Ig (0.3 µg/ml) with anti-ERMAP mAb (2.5, 5, 10 µg/ml) or isotype Ab (10 µg/ml) for 3 days. [3H] thymidine (1 μCi/well) was added to the cultures 12 h before harvest. T cell proliferation was measured by [3H] thymidine incorporation. Results are expressed as counts per minute (CPM). (B, C) Splenic cells were cultured with anti-CD3 Ab in the presence of control Ig or human ERMAP-Ig (0.3 µg/ml) with anti-hERMAP mAb or control Ab (5 µg/ml). T cells were analyzed for the expression of CD69 24 h later. **B** Representative flow cytometric and **C** statistical analysis of the percentage of CD69^+^cells in CD4 and CD8 T cells. **D**, **E** Macrophages were generated from BM of C57BL/6 mice and cultured with HiLyte Fluor 647-Aβ42 in the presence of anti-hERMAP mAb or isotype Ab (5, 10 µg/ml) for 2 h. **D** Representative flow cytometric profiles and **E** statistical analysis for the percentage of HiLyte Fluor 647-Aβ42^+^ cells in F4/80^+^ macrophages. The data are expressed as mean + SD and representative of 3 independent experiments with similar results. *P < 0.05 compared with isotype Ab

We first produced anti-ERMAP mAbs by immunizing BALB/c mice with human ERMAP-Ig protein. The splenocytes were fused to X63-Ag8.653 myeloma cells to produce hybridomas. ELISA was performed to identify hybridomas secreting mAbs which bound to the ERMAP-Ig fusion protein. We then screened the mAbs to assess their ability to neutralize the inhibitory activity of ERMAP on T cells in vitro. Consistent with our previous data, ERMAP-Ig protein inhibited anti-CD3 antibody (Ab) induced T cell proliferation (Fig. 1A) [30]. An anti-ERMAP mAb (clone D4-2) neutralized the inhibitory activity of ERMAP-Ig on T cell proliferation, whereas control isotype Ab did not (Fig. 1A). The anti-ERMAP mAb also neutralized anti-CD3 Ab-induced CD69 expression on CD4 and CD8 T cells (Fig. 1B, C).

We have previously reported that anti-ERMAP polyclonal Ab can enhance macrophage phagocytosis of cancer cells [30]. To determine whether the anti-ERMAP mAb can affect macrophages phagocytosing Aβ, bone marrow-derived macrophages were generated as described [16], and incubated with HiLyte Fluor 647-Aβ42 in the presence of the anti-ERMAP mAb or isotype Ab for 2 h. The cells were then analyzed for the percentage of HiLyte Fluor 647^+^ cells in F4/80^+^ macrophages. As shown in Fig. 1D and E, the anti-ERMAP mAb significantly enhanced the ability of macrophage to phagocytose Aβ.

### Anti-ERMAP mAb improves cognitive performance in AD mice

**Fig. 2:**
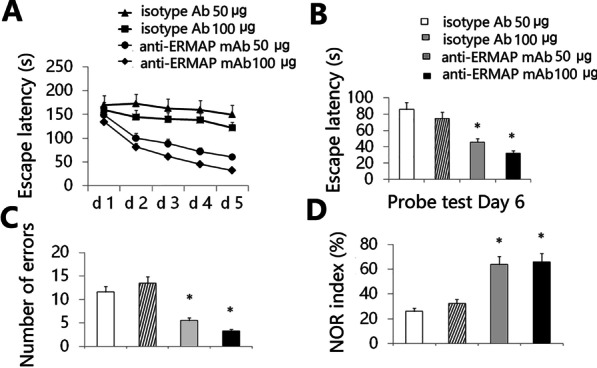
Anti-ERMAP mAb improves cognitive performance in AD mice. Female 3XTg-AD mice (12-month-old) were injected i.p. with anti-ERMAP mAb or isotype Ab once a week. Three months later, the mice were evaluated for cognitive performance by Barnes Maze and NOR tests. **A** The escape latency during the training period, and **B** and **C** the escape latency and the number of errors committed during the probe trial are shown. **D** NOR index was determined as the time spent interacting with the novel object divided by the total time of exploration during the testing phase. The data are expressed as mean ± SD from one of three independent experiments with similar results (5 to 8 mice per group in each experiment). *P < 0.05 versus isotype Ab group

We determined whether administration of anti-ERMAP mAb affected cognitive performance in AD mice. 3XTg-AD mice aged 12 months, an age of advanced cerebral pathology, were injected intraperitoneally (i.p.) with anti-ERMAP mAb or isotype Ab (50 µg or 100 µg per mouse) once a week for 3 months. The mice were then evaluated for spatial learning and memory. It has been shown that the Barnes maze, a hippocampal-dependent spatial task [36, 37], is the most sensitive test for detecting cognitive deficits in 3XTg-AD mice [38]. We found that anti-ERMAP mAb at both doses significantly improved Barnes maze learning curves, as compared with equal dose of isotype Ab (Fig. 2A). Anti-ERMAP mAb-treated AD mice also had decreased latency to find the target zone during the probe trial conducted 24 h after the final training session (Fig. 2B), indicating an improved memory performance. In addition, the number of errors committed in anti-ERMAP mAb-treated mice was also significantly reduced (Fig. 2C).

The NOR test is to assess learning and memory in rodents based on their spontaneous tendency to have more interactions with a novel than with a familiar object [39], representing a more cortically dependent novel object recognition preference task [36, 37]. In agreement with the results in the Barnes maze task, anti-ERMAP mAb-treated mice performed significantly better than control-treated mice (Fig. 2D). Collectively, our results suggest that administration of anti-ERMAP mAb improves spatial learning and memory in AD mice.

### Anti-ERMAP mAb-treated AD mice have reduced AD pathology

**Fig. 3:**
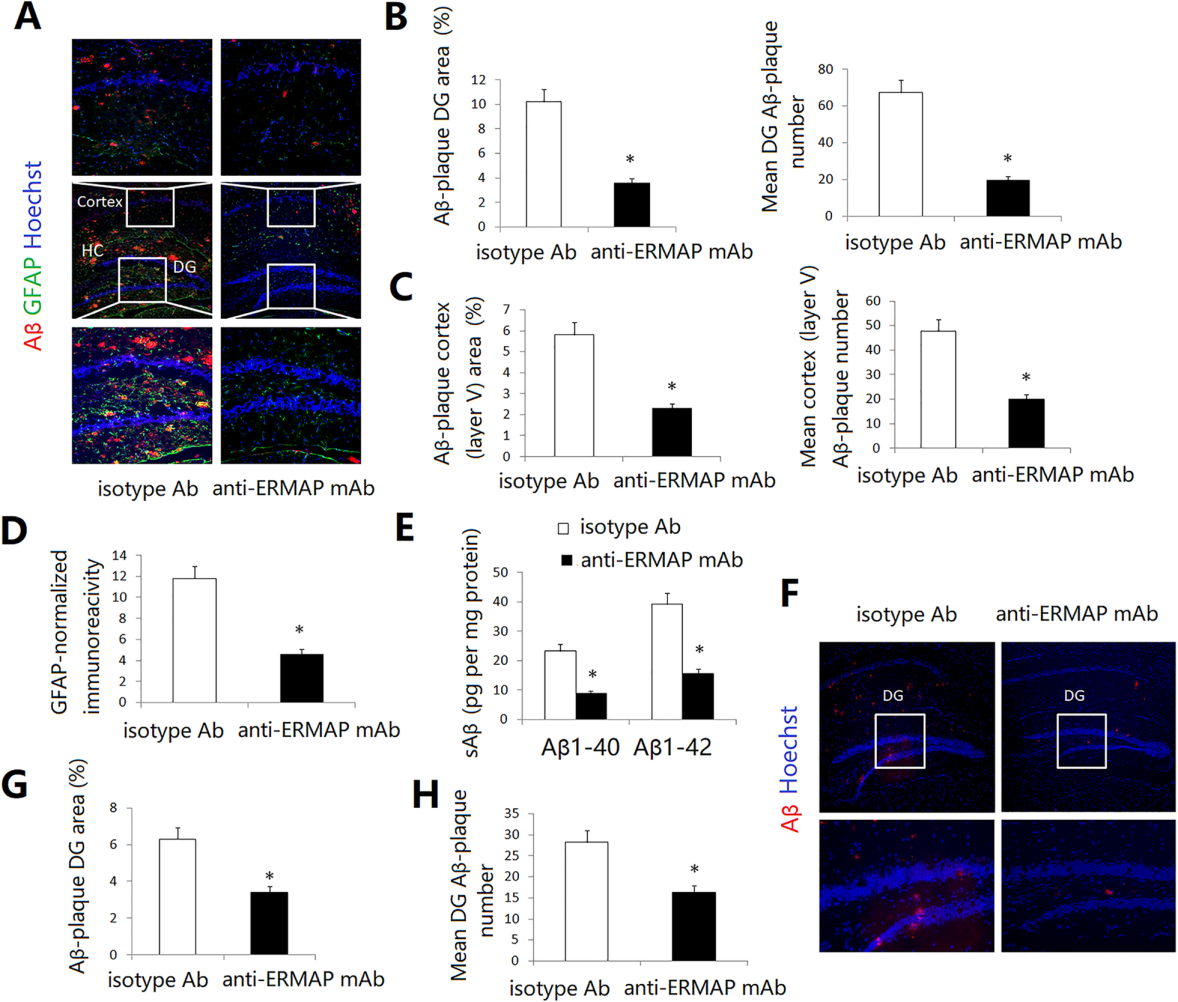
Administration of anti-ERMAP mAb attenuates AD pathology. **A**–**C** 3XTg-AD mice were injected with anti-ERMAP mAb, or control isotype Ab (50 µg per mice) once a week. Three and a half months later, the mice were examined for **A**–**D** brain pathology, and **E** soluble Aβ levels. The brain sections were immunostained for Aβ (in red), GFAP (in green) and Hoechst nuclear staining. Mean Aβ area and plaque numbers in the hippocampal DG and the cortex fifth layer, and GFAP immunoreactivity in the hippocampus were assessed. **A** Representative immunofluorescent images, and **B**–**D** quantitative analysis of Aβ and GFAP. **E** Levels of soluble Aβ1–40 and Aβ1–42 in the cerebral brain parenchyma of the mice were quantified by ELISA. **F**, **G** 14-month-old female APP/PS1 mice were injected with anti-ERMAP mAb, or isotype Ab (50 µg) once a week. Two months later, the mice were evaluated for brain pathology. **F** Representative immunofluorescent images, and **G** quantitative analysis of Aβ. The data are expressed as mean ± SD from one of three independent experiments with similar results (5 to 8 mice per group per experiment). *P < 0.05 versus control group

We then evaluated the ability of anti-ERMAP mAb to improve AD pathology. After the Barnes maze and NOR tests (Fig. 2), the brains were harvested and analyzed by immunohistochemistry. Anti-ERMAP mAb-treated 3XTg-AD mice had a reduced cerebral Aβ plaque load in the hippocampus (HC) [such as the dentate gyrus (DG) and CA1] and in the cerebral cortex (such as layer V) areas (Fig. 3A–C and data not shown). Astrogliosis, as assessed by glial fibrillary acid protein (GFAP) immunoreactivity, was also reduced in anti-ERMAP mAb-treated mice, as compared to control Ab-treated mice (Fig. 3A, D).

Since impaired synaptic plasticity and memory deficits in AD are related to elevated cerebral soluble Aβ1-40/Aβ1-42 (sAβ) levels [40], we measured sAβ levels in the AD mice by ELISA. Consistent with the immunohistochemical results, anti-ERMAP mAb-treated mice had reduced cerebral sAβ, as compared to control-treated mice (Fig. 3E).

We determined the effect of anti-ERMAP mAb in another AD model, APP/PS1 mice, which develop Aβ-plaque pathology at a more advanced age than do 3XTg-AD mice. Administration of anti-ERMAP mAb also reduced hippocampal Aβ plaque load, as compared to control-treated mice (Fig. 3F, G). Taken together, our results suggest that administration of anti-ERMAP mAb into AD mice leads to clearance of Aβ plaques and reversal of cognitive decline.

### Reduced Aβ load in anti-ERMAP mAb-treated mice is not a result of decreased amyloid precursor protein (APP) expression or Aβ production

**Fig. 4:**
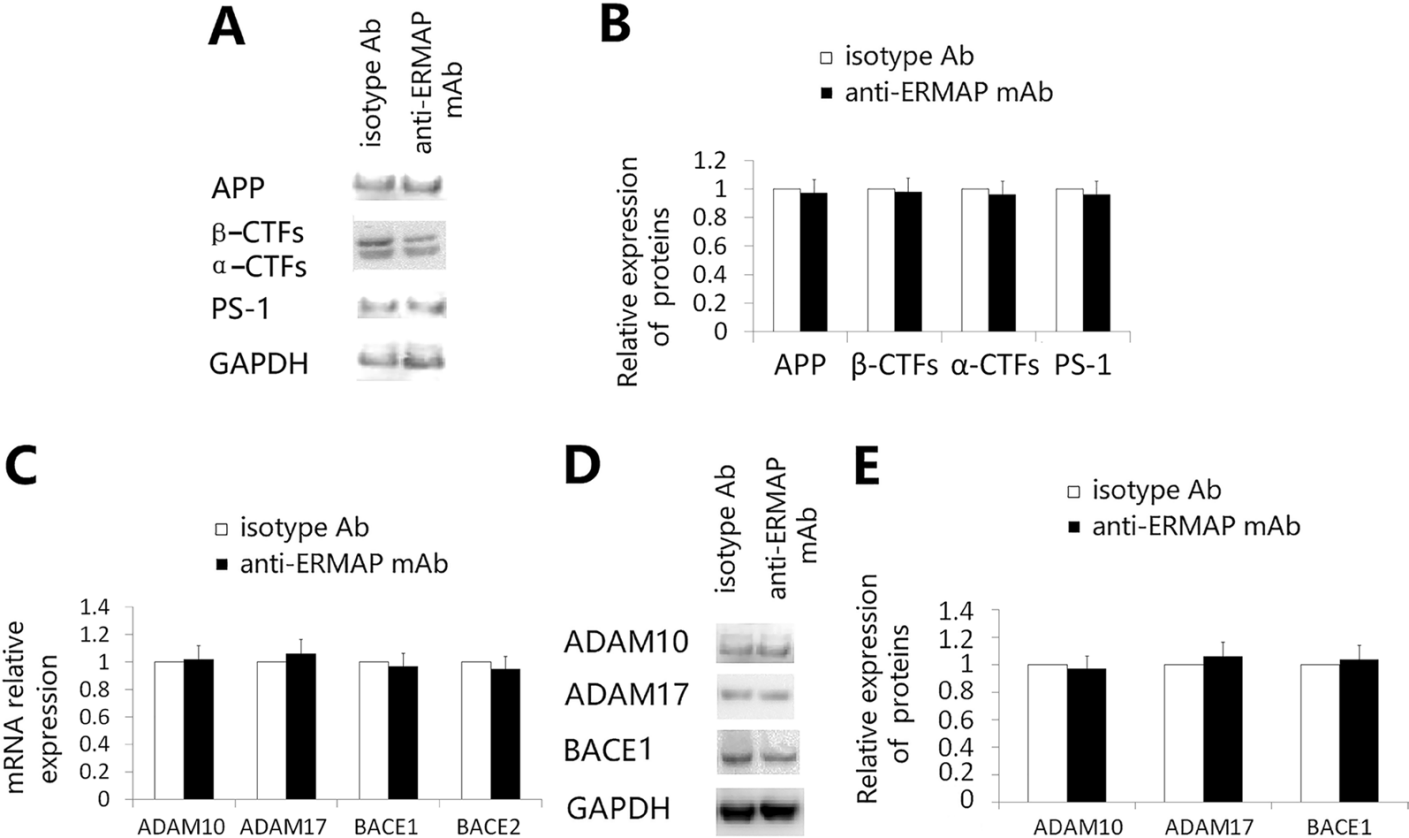
Anti-ERMAP mAb does not alter the expression of APP expression and APP-processing enzymes. 3XTg-AD mice were injected with anti-ERMAP mAb, or control isotype Ab as in Fig. 3. Three and a half months later, the brains were harvested. **A**, **B** The expression levels of APP and PS-1 proteins as well as the APP cleavage products β-CTF and α-CTF were analyzed by Western blot. GAPDH was used as a loading control. **A** Representative Western blot images, **B** quantification of the expression levels of APP and PS-1 proteins. **C** qRT-PCR analysis of the relative expression levels of the genes for the APP-processing enzymes ADAM10, ADAM17, BACE1, and BACE2. **D**, **E** Western blot analysis of the expression levels of ADAM10, ADAM17 and BACE1 proteins. **B**, **C**, **E** The expression level of the protein or gene in control Ab-treated AD mice is defined as 1. The data are expressed as mean ± SD from one of three independent experiments with similar results (5 to 8 mice per group in each experiment)

Since administration of anti-ERMAP mAb significantly reduces Aβ plaque load, we sought to determine whether these findings are due to reduced Aβ production or increased clearance. We first examined the protein levels of APP and PS-1 by Western blot [17]. No differences in the expression of APP and PS-1 as well as the APP cleavage products β-CTF and α-CTF were observed between anti-ERMAP mAb and control isotype Ab-treated AD groups (Fig. 4A, B). Furthermore, the expression levels of the key APP processing genes and proteins ADAM10, ADAM17, BACE1, and BACE2 were not significantly different between anti-ERMAP mAb and control Ab-treated AD mice as measured by qRT-PCR and Western blot (Fig. 4C–E). The results suggest that the observed reduction in Aβ plaque load in anti-ERMAP mAb-treated AD mice is not due to decreased APP production and/or processing, but rather likely mediated via increased Aβ clearance.

### Anti-ERMAP mAb-treated AD mice have increased proportion of T cells and enhanced choroid plexus (CP) activity

**Fig. 5:**
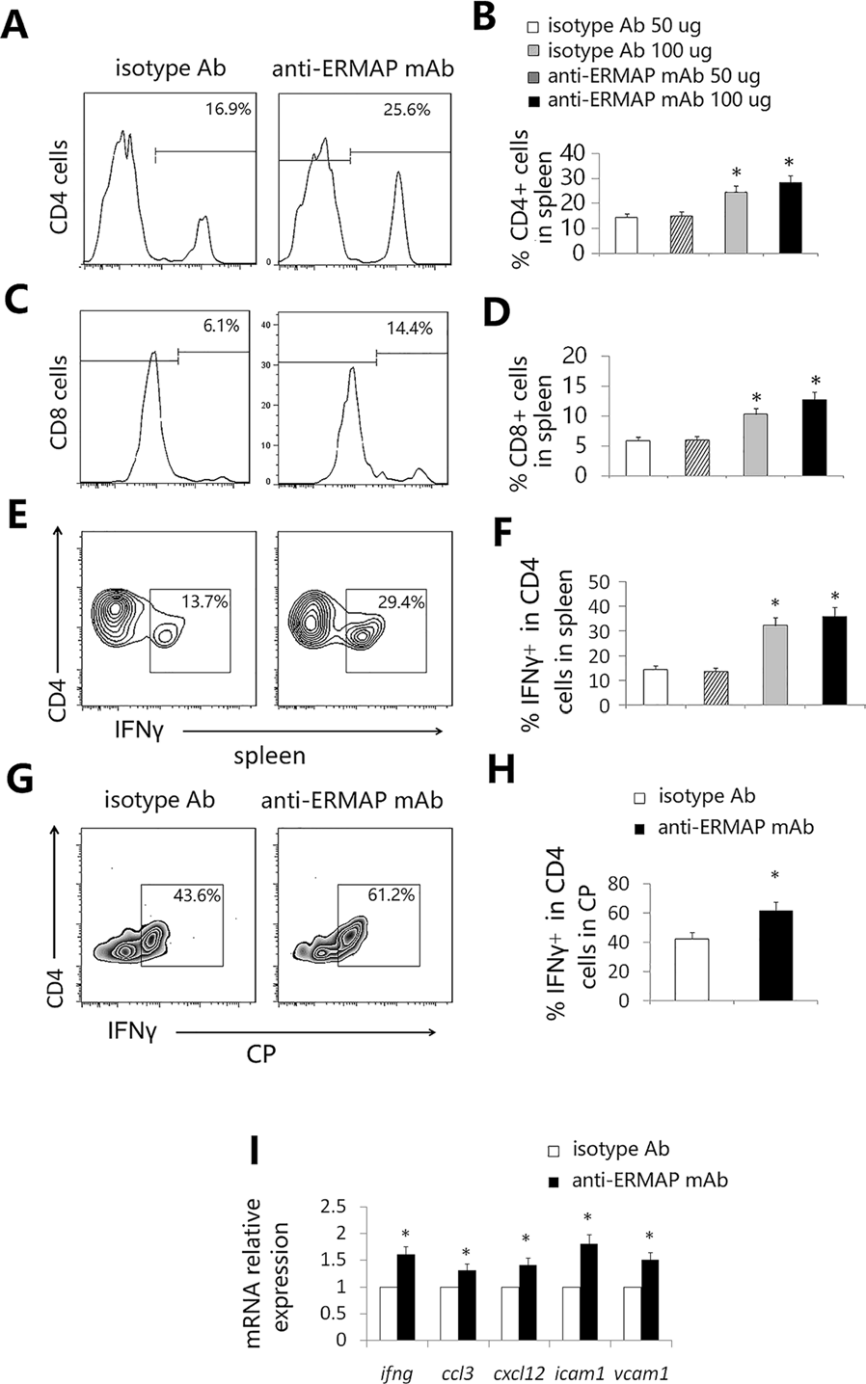
Anti-ERMAP mAb increases the proportion of T cells and the CP activity. 3XTg-AD mice were injected with anti-ERMAP, or control mAb as in Fig. 3. Three and a half months later, the spleen and CP were harvested. The splenocytes were analyzed for the percentages of **A**–**D** CD4 and CD8 T cells, and **E**, **F** IFN-γ-producing CD4 T cells by flow cytometry. The CP was analyzed for **G**, **H** the percentage of IFN-γ-producing CD4 T cells by flow cytometry, and **I** the mRNA expression levels of IFNγ, ccl3, cxcl12, icam1, and vcam1 by qRT-PCR. The expression levels of the genes in control mAb-treated AD mice are defined as 1. The data are expressed as mean ± SD from one of three independent experiments with similar results (5–8 mice per group in each experiment). *P < 0.05 versus control mAb group

We have previously demonstrated that ERMAP protein inhibits T cell proliferation [16]. Since the anti-ERMAP mAb can neutralize the T cell inhibitory activity of ERMAP, we analyzed T cells in the anti-ERMAP mAb-treated mice. As shown in Fig. 5A–D, anti-ERMAP mAb significantly increased the percentages of both CD4 and CD8 T cells in the spleen.

It has been reported that anti-PD-L1/PD-1 Ab reduced AD pathology involves an IFNγ-dependent immunological response [5]. We also analyzed IFNγ-producing T cells in the spleen and found that the percentage of IFNγ-producing CD4 T cells in anti-ERMAP mAb-treated AD mice was significantly higher than that in control mAb-treated mice (Fig. 5E, F).

The CP, the epithelial layer that forms the blood–CSF barrier, is a selective gateway for leukocyte entry to the brain [22]. It has been reported that AD mice have a defect in the CP gateway, as indicated by significantly lower levels of immune cells trafficking molecule expression in the CP [22]. In contrast, IFNγ signaling enhances the expression of the trafficking molecules [41]. We examined IFNγ availability at the CP. Flow cytometric examination showed a significantly higher percentage of IFNγ-producing CD4^+^ immune cells in the CP in anti-ERMAP mAb-treated AD mice (Fig. 5G, H). qRT-PCR analysis confirmed a higher IFNγ mRNA expression level in this compartment of anti-ERMAP mAb-AD mice (Fig. 5I).

Since increased IFNγ availability can enhance CP activity [5, 22], we also analyzed the mRNA expression levels of a panel of immune cell trafficking molecules by qRT-PCR. We found that the expression levels of the genes for chemokine C-C motif ligand 3 (ccl3), and C–X–C motif chemokine 12 (cxcl12), intercellular adhesion molecule 1 (icam1) and vascular cell adhesion molecule 1 (vcam1) in the CP of anti-ERMAP mAb-treated mice were significantly higher than those in control-treated mice (Fig. 5I). Taken together, our results suggest that administration of anti-ERMAP mAb results in increased percentage of T cells, especially IFNγ-producing T cells in the spleen and the CP, and enhanced CP activity.

### Anti-ERMAP mAb-treated AD mice have increased levels of anti-Aβ Abs in the serum

**Fig. 6:**
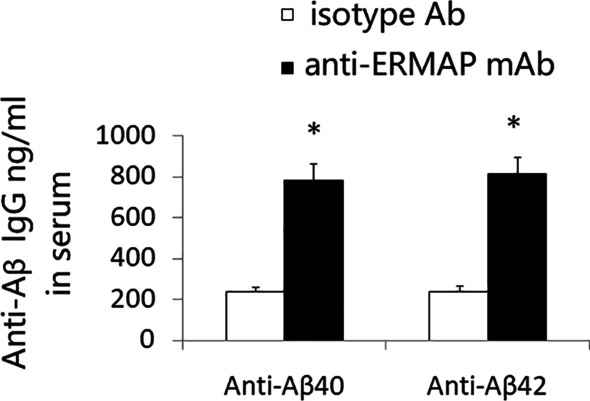
Anti-ERMAP mAb-treated AD mice have increased levels of anti-Aβ Abs in the serum. 3XTg-AD mice were injected i.p. with anti-ERMAP and control isotype Ab as in Fig. 3. Three and a half months later, the levels of anti-Aβ40 and anti-Aβ42 Abs in the serum were measured by ELISA. The data are expressed as mean ± SD from one of three independent experiments with similar results (5–8 mice per group in each experiment). *P < 0.05 versus control isotype Ab group

It is well known that T cells can help B cell to produce antibodies. We analyzed the levels of anti-Aβ Abs in the serum of anti-ERMAP mAb-treated AD mice. The levels of both anti-Aβ40 and anti-Aβ42 Abs in the serum of anti-ERMAP mAb-treated mice were higher than those in control-treated mice (Fig. 6). The results suggest anti-ERMAP mAb treatment also increases the production of anti-Aβ Abs.

### Anti-ERMAP mAb increases the proportion of monocyte-derived macrophages in the brain and the spleen, and the macrophages have enhanced Aβ phagocytosing ability

**Fig. 7:**
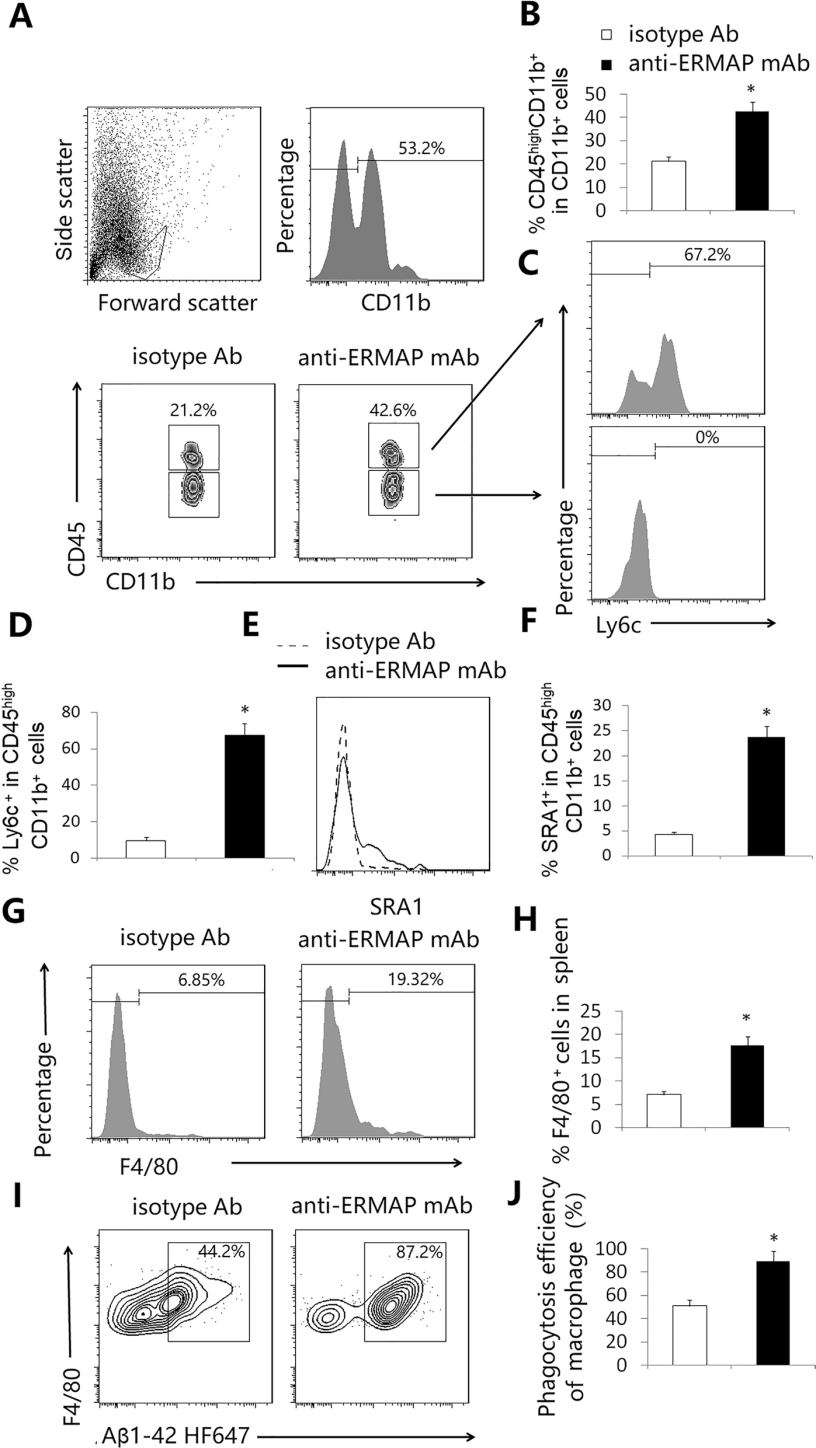
Anti-h ERMAP mAb increases the proportion and the function of macrophages. 3XTg-AD mice were injected with anti-ERMAP mAb, or control isotype Ab as in Fig. 3. Three and a half months later, the brain and the spleen were harvested. The brain was analyzed for the **A**, **B** percentage of CD45^hi^CD11b^+^ cells, **C** the expression of Ly6C by CD45^hi^ or CD45^lo^ cells, **D** the percentage of Ly6C^+^ in CD45^hi^CD11b^+^ cells, and **E**, **F** the expression of SRA1 by CD45^hi^CD11b^+^ cells. The splenocytes were analyzed for **G**, **H** the percentage of F4/80^+^ macrophages, and **I**, **J** the macrophages were analyzed for the ability to phagocytose HiLyte Fluor 647-Aβ42. The data are expressed as mean ± SD from one of three independent experiments with similar results (5–8 mice per group in each experiment). *P < 0.05 versus control Ab group

It has been reported that increased CP activity can result in recruitment of monocyte-derived macrophages to the brain to attenuate AD pathology [5, 22]. Since anti-ERMAP mAb treatment increases CP activity, we examined whether there was an increased proportion of monocyte-derived macrophages in the brain. CD45^hi^CD11b^+^ cells represent a myeloid population enriched with CNS-infiltrating monocyte-derived macrophages in the brain [5, 22]. We found anti-ERMAP mAb-treated AD mice had an elevated percentage of CD45^hi^CD11b^+^ cells in the brain, as compared to control-treated mice (Fig. 7A, B). The CD45^hi^CD11b^+^ cells in anti-ERMAP mAb-treated mice also had a higher percentage of lymphocyte antigen 6c (Ly6C) positive cells than control-treated mice (Fig. 7C, D). In addition, the CD45^hi^CD11b^+^ cells in anti-ERMAP mAb-treated mice expressed a higher level of scavenger receptor A (SRA1) (Fig. 7E, F).

We also analyzed macrophages in the spleen and found the percentage of F4/80^+^ macrophages in anti-ERMAP mAb-treated AD mice was higher than that in control-treated mice (Fig. 7G, H). Furthermore, macrophages in anti-ERMAP mAb-treated mice were more able to phagocytose Aβ42 than those in control-treated mice (Fig. 7I, J). The results suggest that anti-ERMAP mAb increases the production and function of macrophages.

## Discussion

In this paper, we have demonstrated that administration of anti-ERMAP mAb into AD mice increases the proportion of T cells, especially IFNγ-producing T cells, in the spleen and the CP. Anti-ERMAP mAb also enhances the expression of several immune cell trafficking molecules in the CP and increases the migration of monocyte-derived macrophages into the brain. Furthermore, anti-ERMAP mAb enhances the ability of macrophages to phagocytose Aβ and the production of anti-Aβ Abs in the serum.

Although autosomal-dominant AD is characterized primarily by mutations that increase production of Aβ or Aβ42/40 ratio [42, 43], sporadic AD patients primarily accumulate Aβ as a result of impaired clearance [44–46]. Our results have shown that there is no change in the expression levels of the APP, PS-1, and the key APP processing molecules between anti-ERMAP and control Ab-treated AD mice, suggesting that the reduced Aβ plaque load in anti-ERMAP mAb-treated AD mice is not due to decreased APP production and/or processing, but rather likely mediated via increased Aβ clearance. It has been reported that hippocampus and cerebral cortex are the main anatomical regions with robust Aβ-plaque pathology in AD mice [47]. The anti-ERMAP mAb reduced cerebral Aβ plaque load in the hippocampus (such as the DG and CA1) and in the cerebral cortex (such as layer V) areas.

T cells are the major component of the adaptive immune system. Although the role of T cells in AD development is a subject of debate, multiple lines of evidence have suggested that T cells play an important role in the CNS maintenance and repair. For example, blocking the T cell inhibitory PD-L1/PD-1 pathway leads to reduced AD pathology [5, 48]. T cell deficiency is associated with increased neuronal loss in animal models of CNS injury or AD [17, 49–52]. In contrast, transplantation of T cells reduces AD pathology [17–20]. Systemic T cells not only participate in CNS repair, but are also needed for life-long brain plasticity [11, 52, 53]. Our results support that T cells have beneficent effect on AD.

It has been reported that CD4 T cells are essential in the activation of B cells to secrete antibodies to mediate humoral immune responses [54, 55]. In AD, the production of anti-Aβ Abs can neutralize the toxicity of Aβ. We have shown that the production of anti-Aβ Abs in the serum of anti-ERMAP mAb-treated AD mice was increased. This may be due to increased generation of anti-Aβ-producing B cells through the help of T cells. Whether anti-ERMAP mAb indirectly acts on B cells through T cells could be answered by future studies deleting T cells in the mice. Since B cells also express the ERMAP receptor [16], it is also possible that the anti-ERMAP mAb neutralizes the possible inhibitory effect of ERMAP on B cells and enhances the functions of B cells to produce anti-Aβ Abs.

Monocytes and macrophages are the central cells of the innate immune system. Bone marrow-derived monocytes can differentiate into macrophages [56]. The recruitment of monocyte-derived macrophages to the brain [15, 49] can lead to reduced AD pathology [5, 8, 21–28, 30] by removing misfolded proteins including Aβ and Tau [21, 57, 58], balancing the local inflammatory milieu [24, 58], reducing gliosis [59], and protecting synaptic structures [24, 60, 61]. We have shown that administration of anti-ERMAP mAb increases the proportion of monocyte-derived macrophages in the brain. It has been reported that IFNγ increases the expression of a number of immune cell trafficking molecules in primary CP cells in vitro, and that IFNγ^−/−^ or IFNγ receptor^−/−^ mice have lower expression levels of these molecules than their wild-type counterparts [41]. IFNγ-blocking antibody also neutralizes PD-1 blockade-induced expression of these molecules in the CP of AD mice [5]. We have shown that administration of anti-ERMAP mAb results in increased proportion of IFNγ-producing T cells, and increased expression of IFNγ, ccl3, cxcl12, icam1 and vcam1 in the CP. It is possible that the increased expression of IFNγ and the immune cell trafficking molecules are responsible for the enhanced migration of the monocyte-derived macrophages in the brain.

In addition to increase proportion of macrophages in the brain and the spleen, we have also demonstrated anti-ERMAP mAb enhances the ability of macrophages to phagocytose Aβ. This is consistent with our previous report that anti-ERMAP Ab enhance macrophage phagocytosis of cancer cells [16]. The results in the current studies have also shown that the monocyte-derived macrophages in anti-ERMAP mAb-treated mice have a higher expression of Ly6C and SRA1. It has been reported that only Ly6C^hi^ monocyte-derived microglia are more able to phagocytize and eliminate soluble Aβ [62] and that Ly6C is also related to neuroprotection [63, 64]. SRA1 is an Aβ-binding scavenger receptor associated with Aβ-plaque clearance [65]. The mechanisms by which anti-ERMAP mAb increase the function of macrophages remain to be determined. It is possible that anti-ERMAP mAb neutralizes the macrophage inhibitory activity of ERMAP and/or enhance T cell functions to help the activation of macrophages. Again, deletion of T cells in anti-ERMAP mAb-treated AD mice may answer the question whether anti-ERMAP mAb indirectly acts on macrophages through T cells. It is well known that brain resident microglia also play an important role in clearing cerebral Aβ deposits. It remains to be determined whether anti-ERMAP mAb affects brain resident microglia.

It has been reported that female 3xTg-AD and APP/PS1 mice have more aggressive Aβ pathology than male mice [66, 67]. We used female AD mice in the studies for this manuscript. However, we have observed a similar trend that anti-ERMAP mAb treatment reduces Aβ pathology and regulates immune cells in male 3xTg-AD and APP/PS1 mice (data not shown).

## Conclusions

We have demonstrated that administration of anti-ERMAP mAb attenuates AD pathology and improves cognitive performance. Blocking the ERMAP pathway has the potential to provide a novel approach to treat AD patients. Anti-ERMAP mAb also provides a novel approach to study the role of immune cells and molecules in AD development.

## Data Availability

The datasets generated during and/or analyzed during the current study are available from the corresponding author on reasonable request.

## Abbreviations

Aβ: Beta-amyloid
AD: Alzheimer’s disease
ERMAP: Erythroid membrane-associated protein
Ab: Antibody
mAb: Monoclonal antibody
CFA: Complete Freund's adjuvant
IFA: Incomplete Freund's adjuvant
CP: Choroid plexus
RT-PCR: Reverse transcription polymerase chain reaction
qRT- PCR: Real-time qualitative RT-PCR
sAβ: Soluble Aβ protein
NOR: Novel object recognition
CPM: Counts per minute
i.p.: Intraperitoneally
HC: Hippocampus
DG: Dentate gyrus
GFAP: Glial fibrillary acid protein
APP: Amyloid precursor protein
PS-1: Presenilin-1
ccl3: C–C motif ligand 3
cxcl12: C–X–C motif chemokine 12
icam1: Intercellular adhesion molecule 1
vcam1: Vascular cell adhesion molecule 1
Ly6C: Lymphocyte antigen 6c
SRA1: Scavenger receptor A
CTFs: C-terminal fragments
